# Supplementary material for: When effort pays off: An experimental investigation into action orientation and anxiety as buffering factors between anhedonia and reward motivation
Source: PLoS One. 2025 Mar 31;20(3):e0320052. doi: 10.1371/journal.pone.0320052 (PMC11957302; doi:10.1371/journal.pone.0320052)
Supplement: Attachment — Submitted filename: ResponseToReviewers.docx [file pone.0320052.s002.docx]

Dear Editorial Staff of PLOS One

Thank you for the opportunity to revise and resubmit this manuscript for PLOS One. We appreciate the valuable comments provided by the expert reviewer and the editor. Please see below for our detailed responses to reviewer comments, which we believe greatly strengthened the quality of the manuscript. We made minor changes throughout the manuscript for grammar, clarity, and conciseness. We also edited the format of the manuscript to ensure it meets the journal’s formatting guidelines.

_____________________________________________________________________________

**Reviewer #1: Gallagher et. al. investigate how action orientation and anxiety moderate the association between anhedonia and reward motivation, as measured by the EEfRT task. The study aims to test two hypotheses: (1) whether, in the absence of stress manipulation, action orientation mitigates the decrease in reward motivation at low levels of anhedonia, and (2) whether the effect of anhedonia on reward motivation depends on the level of anxiety.**
**The manuscript addresses an important and complex topic, employing computational modeling to examine nuanced relationships between key variables. The finding that action orientation buffers diminished reward motivation at high levels of anhedonia is particularly intriguing and provides valuable insights into individual differences in reward-related behaviors. However, several aspects of the manuscript could benefit from refinement to enhance clarity, organization, and coherence. Below, I outline major and minor points for consideration.**

Thank you for taking the time to review our manuscript and for providing helpful comments. Please see below for our point-by-point responses and changes to your suggested edits.

Major Points

**1. Readability and Clarity**
**The manuscript could benefit significantly from improved readability. Many sentences are excessively long, with numerous commas and parentheses, making the text difficult to follow. Additionally, some sentences contain redundant information that could be omitted without altering their meaning. Streamlining these sentences would improve flow and accessibility.**
**For instance:**
**Lines 7-14 of the introduction describe the relationship between anhedonia, reward-seeking behavior, and reward motivation. The first sentence introduces the concept, while the second and third sentences reiterate the same ideas with minor variations. This redundancy detracts from the flow and clarity of the argument.**
**Similarly, lines 15-21 of the introduction (continued on the first two lines of page 4) describe a cycle in which anhedonia leads to reduced positive affect and motivation, which in turn reinforces anhedonia. While this cycle is important, the text does not explain what might initiate or perpetuate it, leaving the reader with unanswered questions.**
**I recommend revising these sections for conciseness and coherence.**

Thank you for pointing out this need for clarification. We have removed and combined sentences for conciseness, while also adding information about the devaluative cycle to improve the flow of this section on pgs. 3-4.

“Prior work has shown that anhedonia is related to decreases in reward-seeking behaviors, specifically the motivation to achieve or ‘want’ rewards (i.e., reward motivation) and decision-making for reward-related tasks or behaviors (5-9). Thus, if one is unable to experience the pleasure or reinforcing nature of apparent rewards, it will likely result in further inhibition or avoidance of the reward in the future.”

“This devaluative cycle of diminished positive affect may be the result of learned emotionally salient experiences. Specifically, Reward Devaluation Theory (i.e., RDT) posits that this reduced motivation for reward may result from repeated pairings of positivity with negative outcomes (4). This learned association may lead to an avoidance of positivity and rewards, as some individuals with depression may experience negative affect in the face of positive stimuli. As such, depressed persons who devalue and avoid positivity likely also experience elevated anhedonia and reduced positive affect, perpetuating this cycle.”

**2. Organization of the Introduction**
**The introduction feels disorganized, with weak logical connections between sentences and vague descriptions of key constructs. For instance:**
**The section on action orientation introduces the term without providing a clear, concise definition. Sentences such as "One potential protective factor..." and "action orientation can be conceptualized as the theoretical inverse of reduced reward motivation..." are verbose and repetitive. Furthermore, the cited source does not define action orientation as the inverse of diminished reward motivation but instead focuses on volitional control and emotional regulation. Clarifying these points and ensuring alignment with cited sources would strengthen the introduction.**
**Additionally, the rationale for removing stress manipulation is not adequately explained, especially given that action orientation is described as a response to stress. A clearer explanation of the study's design choices would help readers understand the context and importance of the findings.**

We have edited this section on pg. 4 to more clearly define action orientation, as well as describe its connection and differences with reward devaluation. We also added a more recent and theoretically relevant review paper of reward devaluation that discusses this theoretical overlap.

“Given the detrimental impact anhedonia can have on personal functioning, is vital to identify factors that may be able to break this devaluative cycle, thus increasing reward motivation. One potential protective factor that may buffer against this diminished reward motivation seen in persons with elevated depression and anhedonia is action orientation, or the ability to upregulate positive affect in the face of stress or negative affect in order to pursue goals (13). Whereas reward devaluation involves avoidance of positive affect due to fear that it will be coupled with negative outcomes, action orientation can be conceptualized as the theoretical inverse given that action orientation involves upregulating positive emotions (or the willingness to approach positivity, rather than avoid it) in the face of negative or stressful events (13, 14).”

We also provided additional rationale for investigating the role of action orientation as a buffer for reward motivation in the absence of a stress manipulation on pg. 8.

“However, given the potential utility of action orientation as a buffer against diminished reward seeking behaviors, additional effort to potentially replicate this finding without a stress manipulation would provide further implications of the role of action orientation. For example, if baseline action orientation, or the tendency to upregulate positive affect when facing challenges, is able to increase reward motivation even when stress in not concurrently being encountered, this would provide evidence for action orientation as a chronically accessible protective factor. Generalizing the role of action orientation as a buffering mechanism for reward motivation regardless of the situational context would provide support for targeting and building action orientation tendencies through future treatments.”

**3. Role of Anxiety**
**The section discussing anxiety's role in reward motivation is somewhat ambiguous. At times, the text suggests that anxiety might buffer reward behaviors (e.g., "anxiety may work as a buffer to increase reward behaviors"), while at other points, it posits that anxiety and anhedonia have similar effects in diminishing reward motivation. These conflicting conclusions are not reconciled, leaving the reader uncertain about the primary hypothesis.**
**To address this, I suggest:**
**Providing a theoretical framework that explains why anxiety might have different effects in different contexts.**
**Clarifying the hypotheses and presenting evidence more cohesively.**
**Including specific, testable hypotheses for future research to resolve these mixed findings.**

Additional theoretical framework, including additional studies/citations, has been included in the background of anxiety and reward motivation on pgs. 6-7. We include these additional studies to motivate our largely exploratory analyses with regard to anxiety, given the mixed literature.

“For example, the impact of anxiety on reward seeking behaviors remains mixed. Although anxiety has been associated with diminished reward motivation (18), other work examining the association between anhedonia and reward-learning behaviors concluded that anxiety was not associated with diminished reward learning abilities (19, 20). In previous work utilizing a different effort-based experiment, Mehta and colleagues found unique motivational differences between those with depression and anxiety based on potential rewards and losses (21). This finding is partially supported by prior work that has investigated the role of anhedonia and anxiety on an emotional attention task (i.e., dot-probe task): anhedonia was associated with slower reaction times on positive valence trials, but was associated with faster reaction times when anxiety was elevated (22). Another study concluded that social anxiety was not characterized by blunted positive affect (23): individuals with social anxiety did not exhibit differential responsivity to reward via behavioral tasks or via neuroimaging, as there was no blunted activity in the ventral striatum, an area involved in reward processing. In fact, socially anxious individuals displayed increased brain activity in the default mode network during both reward anticipation and consummation. Thus, it is possible that reward motivation may not be diminished in those with social anxiety, but may involve hypervigilance and fixation on self-performance when in the presence of prospective reward.”

We also added clarity to the current study section on pg. 9, as the anxiety model was largely explorative given the mixed literature.

“As it remains unclear whether anxiety may further decrease the low reward motivation that is seen in anhedonia, or whether the hypervigilance and physiological arousal that accompanies anxiety would act as a slight potential buffer, we investigated the interactive effect to assess these competing hypotheses. No specific hypotheses for the anxiety model were outlined in the pre-registration, as this largely explorative examination sought to gain clarity with regards to these competing hypotheses. Specifically, we investigated the interactive effect of anhedonia and anxiety, in addition to the main effects, on reward motivation.”

**4. Figures and Visual Presentation**
**The figures are low in quality, and the colors used are difficult to distinguish between conditions. I recommend improving the resolution of the figures and selecting colors that are more discernible for readers.**

We recreated the figures in R to improve image quality and to make the colors of the various groups more discernible. We apologize if the figures still portray less than optimal resolution, as we are also following the journal required format for images (i.e., .tif files).

**5. Missing Data**
**The manuscript states that 25 participants did not complete the action orientation scale, but it is unclear how this missing data was managed in the moderation analysis. Since these models require complete data for the moderator, predictor, and outcome variables, it seems likely that the sample size was effectively reduced to 75. However, the manuscript implies that all 100 observations were used. Please clarify how missing data were handled, discuss any potential issues arising from the unevenness, and explain how these issues were addressed.**

Thank you for pointing out this need for clarification. We have included additional language throughout the manuscript, including in each model’s results section, to reflect the fact that all analyses involving action orientation had a reduced sample size of 75, whereas we used all available data for the anxiety moderator analyses. Since the 25 participants were not missing data on the BAI, we included all 100 observations in that analysis to maximize power.

“Moreover, the ACS was only assessed at the follow-up session, which was optional and thus included valid and complete responses from 75 participants. Thus, in an effort to use all available data to maximize power and remain consistent with using the same EEfRT data in analyses across models, the EEfRT, anhedonia, and anxiety measures were all completed during the first session, whereas only action orientation was obtained at the follow-up session. This resulted in 75 and 100 complete observations in the action orientation and anxiety models, respectively.”

“Identical to the GEE model, the 75 available observations with complete data on the ACS were utilized to ensure complete data in the moderation analyses.”

“All available 100 observations were utilized in Model 2 to maximize power.”

Minor Points

**6. Title: The title reflects the key variables and experimental approach but could be refined to emphasize the computational modeling aspect and the interaction between variables. This would make it clearer and more informative.**

We have changed the title to reflect the interaction between the included variables, as well as to account for the potential buffering effect:

“When effort pays off: An experimental investigation into action orientation and anxiety as buffering factors between anhedonia and reward motivation.”

**7. Participant Compensation: There is inconsistency in describing participant compensation. Page 10, lines 20-22 state that participants were paid based on two randomly selected win trials, while page 11, lines 11-13 suggest they were paid the total of all wins. Please clarify which description is accurate. Furthermore, it is stated that at the end of each session participants were provided with local and national referral resources (page 11, last two lines), but no mention of what kind of resources they were referred to.**

We have clarified that compensation was based on the two randomly selected win trials from the EEfRT. We also updated the language to describe that mental health resources were provided to participants following the study on pg. 13.

“At the end of the task, the experimenter noted the total reward from the two randomly selected win trials on the EEfRT that was shown on the screen, which was paid to participants.

At the end of each session, regardless of their responses on the self-report measures or tasks, local and national resources for mental health services were provided to each of the participants.”

**8. EEfRT Task Explanation: Page 5, lines 21-22 describe how reward motivation and effortful behavior were derived from the EEfRT task but do so vaguely. Explicitly defining how these measures were calculated (e.g., low-probability rewards for reward motivation, HC/HR trials for effortful behavior) would eliminate room for speculation.**

We have added language defining the LC/LR and HC/HR trials, as well as the role of probability on pg. 5.

“Participants are presented with the option of engaging in a more difficult task with the prospect of gaining a larger reward (i.e., High Cost/High Reward (HC/HR)) or rather pursuing an easier task with a lower associated reward (i.e., Low Cost/Low Reward (LC/LR)). Each trial includes the probability that the accompanied trial will be a win trial, which factors into the cost-benefit analysis for the participant. In addition to this reward decision making process, participants must also engage in and complete the task, for the opportunity to obtain the associated reward. Whereas the LC/LR task requires participants to complete 30 button presses with their dominant index finger, the HC/HR task requires 100 button presses with the nondominant pinky finger (15).”

**9. Compliance with Task Instructions: Page 10, line 16 mentions task instructions (e.g., using the pinky of the non-dominant hand). Were participants supervised, or were video recordings used to ensure compliance?**

We added the following language on pg. 12 regarding monitoring for task compliance.

“All participants were video recorded while completing the EEfRT, and both undergraduate research assistants and a graduate research assistant monitored the live video during the task to ensure compliance with task instructions.”

**10. Trial Distribution: Page 11, line 10 states that the number of trials was not fixed as participants had to complete as many trials as they could in a 20 minute timespan. How, then, did the authors ensure equal numbers of high, medium, and low-probability trials?**

We now included additional details regarding the randomization of the trial probabilities on the EEfRT on pg. 13.

“Trials were counterbalanced and presented in pseudorandomized order. The trials were fixed in that each participant received the same trials in the same order, but some participants received more trials depending on how quickly they completed each trial. Nevertheless, all participants received approximately an equal number of each trial probability due to counterbalancing, regardless of how many trials were completed. The smallest number of trials completed by a participant was 52 (18 of 12%; 17 of 50%; and 17 of 88% probability trials). The largest number of trials completed by a participant was 186 (61 of 12%, 63 of 50%, and 62 of 88% probability trials).”

**11. Inclusion Criteria: Page 11, line 18 mentions that only participants who attended at least four sessions were included, yet only data from the first session were used in the main analysis. Clarifying the rationale for this criterion would improve transparency.**

We edited this language on pg. 13 to clarify that of the entire 101-person sample, all of the participants had attended at least four of the six weekly sessions.

“All participants attended a minimum of four of the six weekly sessions, and the follow-up session was optional.”

**12. Grammar and Typos: There are minor grammatical errors (e.g., page 3, lines 3-4, where the verb does not agree with the subject) and typos (e.g., page 6, line 7). A careful proofreading would address these issues.**

We have carefully proofread the updated manuscript and corrected identified typos and grammatical errors.

**Reviewer #2: This manuscript, crafted by Gallagher and colleagues, provides an insightful evaluation of the psychological constructs ‘action orientation’ and ‘anxiety’ and their interactions with anhedonia, when modulating reward motivation in the EEfRT task. The two aims of this study were aimed at exploring: 1. that lower intensities of action orientation reflect decreases in reward motivation when anhedonia is present, and 2. that anxiety, depending on intensity, increases or decreases reward motivation in conjunction with anhedonia.**

**1. The present analyses derived from a solid sample of individuals with depressive symptoms reveals that 'action orientation' can protect ‘reward motivation’ when anhedonia is also highly expressed. While the results of the moderation analysis for anhedonia and anxiety on reward motivation were less robust, I did find that the ability of high anxiety to lean-towards-a-buffering-effect to be an important contribution for many disciplines that are concerned with anhedonia, affective states, or reward processing. My only comment for the authors to consider here is that a little more science pertaining to 'anxiety' and it ability to modulate ‘reward motivation’ be provided in the Discussion section.**

Thank you for your valuable feedback on our manuscript. We have added several citations and expanded our interpretation of the anxiety results in the discussion section on pg. 21. We also referred the readers to a recently developed task of reward devaluation that can be used to examine other moderating variables on pg. 23.

“This is partially supported by past work which found differential responsivity to rewards and losses in depressed and anxious groups (21). Although they utilized a different task, individuals with anxiety displayed greater motivation to avoid potential losses than those with depression. Additionally, anxiety was associated with greater extrinsic motivation, compared to the depressed group, during the reward task. The motivation to avoid loss (i.e., the opportunity to gain reward) in the EEfRT paradigm, as well as being motivated by the monetary extrinsic reward in the EEfRT task may be potential factors that contributed to the current findings. Recent work using advanced learning algorithms found that depending on the learning strategy employed, differences in reward decision making emerges based on anhedonia and anxiety severity (35). That is, differences in reward decision making can emerge depending on if the algorithm considers all previous trials and probabilities or just the trial that immediately preceded.”

“A related advance that measures the tendency to devalue reward but has increased ecological validity is the newly introduced Valence Selection Task (VST; 43). Future research examining the interface of willingness to expend effort and reward devaluation can incorporate both the EEfRT and the VST.”
